# Extensive pneumocephalus in a fatal central nervous system infection caused by NDM-1–producing carbapenem-resistant *Klebsiella pneumoniae*: a case report

**DOI:** 10.3389/fmed.2026.1905801

**Published:** 2026-08-25

**Authors:** Chen Zhang, Ye Lu, Jun Ni, Lina Chen, Qi Li, Fangyu Yu, Xiaohong Zhou

**Affiliations:** 1 Intensive Care Unit, Ningbo Municipal Hospital of Traditional Chinese Medicine (TCM), Affiliated Hospital of Zhejiang Chinese Medical University, Ningbo, China; 2 Department of Emergency Medicine, Lanxi People’s Hospital, Lanxi, China; 3 Department of Radiology, The First Affiliated Hospital, Zhejiang University School of Medicine, Hangzhou, China; 4 Department of Critical Care Medicine, Sir Run Run Shaw Hospital, Zhejiang University School of Medicine, Hangzhou, China; 5 Department of Ultrasound, Lanxi People’s Hospital, Lanxi, China

**Keywords:** *bla*NDM-1 gene, central nervous system infection, genomic characterization, multidrug resistance, pneumocephalus

## Abstract

**Background:**

Central nervous system (CNS) infections caused by New Delhi metallo-β-lactamase-1 (NDM-1)-producing carbapenem-resistant *Klebsiella pneumoniae* (CRKP) are rare but associated with extremely high mortality because of extensive antimicrobial resistance and poor blood–brain barrier (BBB) penetration. To the best of our knowledge, there have been no published reports of pneumocephalus associated with infection caused by NDM-1-producing *K. pneumoniae*.

**Case presentation:**

We report an 18-year-old woman who developed bloodstream infection and metastatic CNS infection following severe thoracoabdominal crush injury. Serial cerebrospinal fluid (CSF) cultures repeatedly yielded NDM-1-producing CRKP despite multiple adjustments of antimicrobial therapy. Retrospective whole-genome sequencing demonstrated that blood and CSF isolates belonged to the same clonal lineage carrying the *bla*NDM-1 gene on an IncX3 plasmid, confirming hematogenous dissemination. Serial cranial computed tomography revealed progressive diffuse cerebral edema and extensive pneumocephalus in the absence of skull fracture or neurosurgical intervention. Persistent microbiological failure was mainly attributed to the combination of NDM-1-mediated multidrug resistance and inadequate CNS antibiotic exposure, which ultimately led to the patient’s death.

**Conclusion:**

This case illustrates the devastating clinical course of NDM-1-producing CRKP CNS infection and identifies extensive pneumocephalus as a rare but potentially fatal complication. It emphasizes the importance of early molecular diagnosis, repeated CSF microbiological assessment, optimization of antimicrobial regimens with adequate CNS penetration, and implementation of effective infection-control strategies. The case also highlights the urgent need for novel therapeutic approaches against metallo-β-lactamase-producing pathogens.

## 1 Introduction

Infections caused by multidrug-resistant (MDR) Gram-negative pathogens, particularly carbapenem-resistant *Klebsiella pneumoniae* (CRKP), pose a major challenge in intensive care units because of their increasing global prevalence and limited treatment options (1). CRKP strains producing New Delhi metallo-β-lactamase (NDM), an enzyme capable of hydrolyzing most β-lactam antibiotics, have been increasingly reported worldwide (2). Among the various NDM variants, NDM-1 and NDM-5 are the most commonly identified in China (3).

Central nervous system (CNS) infections are associated with high mortality, particularly when caused by carbapenem-resistant Enterobacteriaceae (CRE) such as *K. pneumoniae*. This poor prognosis is largely attributed to the limited availability of effective antibiotics and the restricted drug penetration across the blood-brain barrier (BBB) (4). Pneumocephalus, defined as the abnormal accumulation of gas within the intracranial cavity, most commonly results from craniofacial trauma but can also occur secondary to infections by gas-producing bacteria. In rare instances, *K. pneumoniae* meningitis may lead to intracranial pneumatosis (5). Despite recent advances in antimicrobial therapy, including the use of novel β-lactam/β-lactamase inhibitor combinations such as aztreonam/avibactam (6), infections caused by NDM-1-producing CRKP often remain refractory due to heteroresistance and suboptimal antibiotic penetration into the CNS (7).

In this case report, we describe a fatal CNS infection caused by NDM-1-producing CRKP with extensive pneumocephalus, aiming to highlight the diagnostic and therapeutic challenges and to underscore the urgent need for novel antimicrobial strategies.

## 2 Case description

An 18-year-old female was admitted to the emergency department in cardiopulmonary arrest following a thoracoabdominal crush injury. She had no history of diabetes mellitus, hypertension, or other underlying medical conditions.

On examination, her temperature was 35.2 °C, heart rate 150 beats per minute, blood pressure 104/47 mmHg. She was apneic and required mechanical ventilation. Pupils were 4 mm and reactive to light. Lung auscultation revealed wet rales and coarse breath sounds bilaterally. The patient remained comatose with a Glasgow Coma Scale (GCS) score of 3 (E1VTM1). Bilateral Babinski signs were absent.

Laboratory examination revealed severe metabolic acidosis combined with respiratory acidosis (pH 6.81, PaCO_2_ 84 mmHg, PaO_2_ 30 mmHg) and hyperlactatemia (12.30 mmol/L). Liver enzymes were elevated: aspartate aminotransferase 716 U/L, lactate dehydrogenase 1403 U/L. Blood glucose was 17.98 mmol/L.

Imaging examination revealed multiple injuries. Bedside emergency ultrasound found a small amount of fluid accumulation in the abdominal cavity with the formation of blood clots. Contrast-enhanced abdominal CT confirmed liver contusion with active bleeding and demonstrated abdominal and pelvic hematoma. Cranial CT revealed subarachnoid hemorrhage (SAH) but no evidence of skull fracture. The three-dimensional CT scan demonstrated fractures of the left 2nd to 5th ribs and possible fractures of the 6th and 7th ribs. Additionally, the pulmonary findings were consistent with acute respiratory distress syndrome.

The patient received therapeutic hypothermia, mechanical ventilation, intracranial pressure (ICP) reduction, and supportive care for multiple injuries. For antibiotic treatment, empiric cefuroxime sodium 1.5 g intravenously every 8 h was started on Day 1. On Day 11, due to rising C-reactive protein (CRP) and procalcitonin (PCT), blood cultures were obtained, and therapy was escalated to imipenem/cilastatin 0.5 g intravenously every 8 h. On Day 14, cultures from blood (collected on Day 11) and an internal jugular venous catheter (collected on Day 14) both grew MDR *K. pneumoniae* (Table 1). On the basis of susceptibility results, treatment was changed to ceftazidime/avibactam 2.5 g intravenously every 8 h in combination with polymyxin B 500,000 U intravenously every 12 h; polymyxin B was discontinued after three days (Day 17), while ceftazidime/avibactam was continued.

**TABLE 1:** Antimicrobial susceptibility profiles of *K. pneumoniae* isolates from different sources and time points.

Antimicrobial Agent	Blood (Day 11)	Catheter (Day 14)	CSF (Day 23)	CSF (Day 35)
Piperacillin-tazobactam	R (≥128)	R (≥128)	R (≥128)	R (≥128)
Ceftazidime	R (≥64)	R (≥64)	R (≥64)	R (≥64)
Ceftriaxone	R (≥64)	R (≥64)	R (≥64)	R (≥64)
Cefepime	R (≥32)	R (≥32)	R (≥32)	R (≥32)
Cefuroxime	R (≥64)	R (≥64)	R (≥64)	R (≥64)
Cefoxitin	R (≥64)	R (≥64)	R (≥64)	R (≥64)
Cefoperazone-sulbactam	R (≥64)	R (≥64)	R (≥64)	R (≥64)
Amoxicillin-clavulanate	R (≥32)	R (≥32)	R (≥32)	R (≥32)
Imipenem	R (≥16)	R (≥16)	R (≥16)	R (≥16)
Ertapenem	R (≥8)	R (≥8)	R (≥8)	R (≥8)
Amikacin	S (≤2)	S (≤2)	S (≤2)	S (≤2)
Trimethoprim-sulfamethoxazole	S (≤20)	S (≤20)	S (≤20)	S (≤20)
Levofloxacin	I (1)	I (1)	S (≤0.12)	I (1)
ESBL	Negative	Negative	Positive	Positive

S, susceptible; I, intermediate; R, resistant. MIC values are expressed in μg/mL. ESBL, extended-spectrum β-lactamase; CSF, cerebrospinal fluid.

**FIGURE 1:**
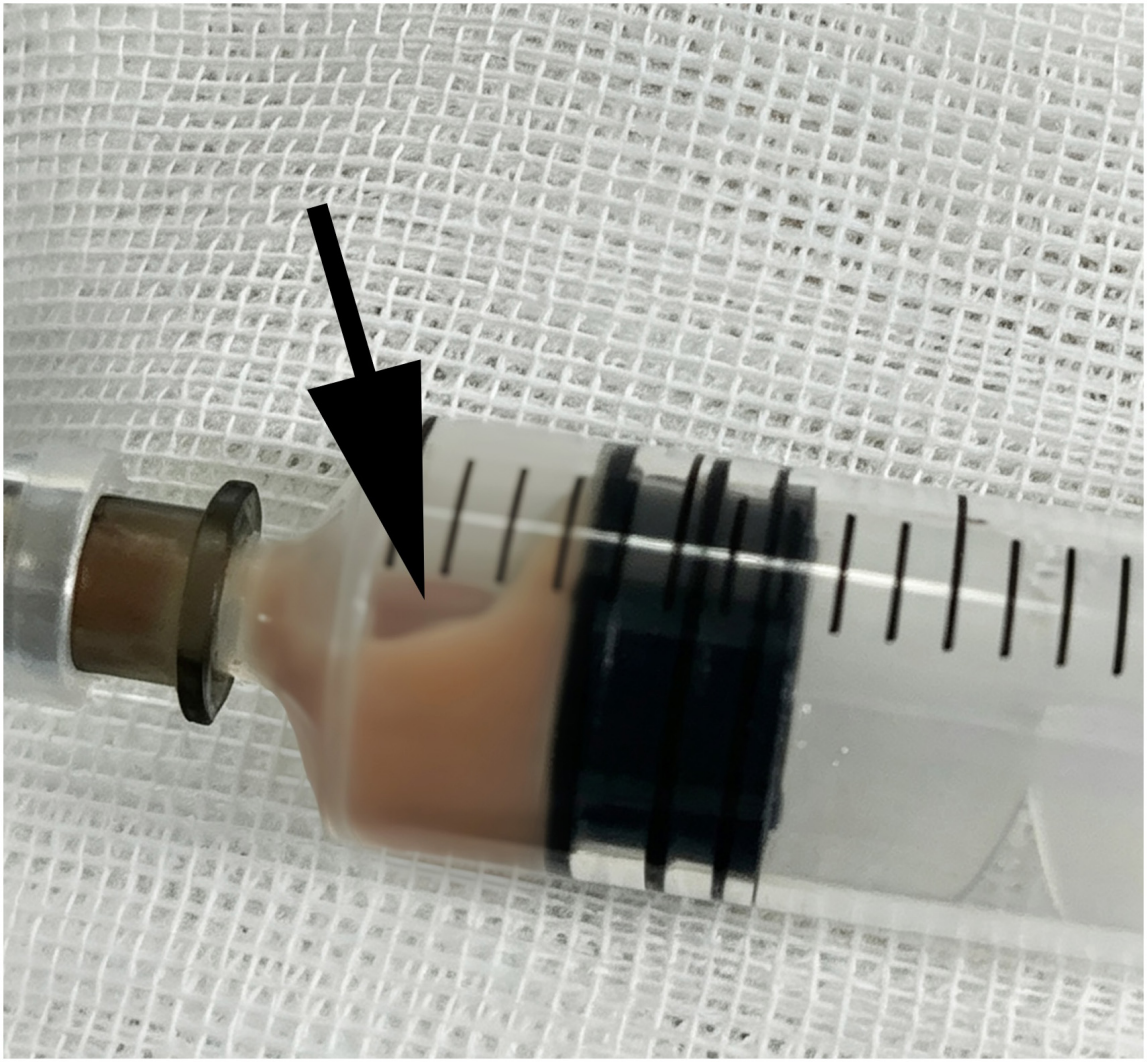
Purulent cerebrospinal fluid (CSF) aspirate. Photograph of the syringe aspirate obtained during the second lumbar puncture on Day 35. The fluid appears frankly purulent and thick (indicated by the black arrow).

On Day 23, a lumbar puncture yielded pale red, turbid cerebrospinal fluid (CSF) with an opening pressure of 250 mmH_2_O. CSF analysis showed a markedly elevated total nucleated cell count of 29,427 cells/μL, with 75% polymorphonuclear cells and 25% mononuclear cells. Biochemical analysis revealed profound abnormalities: glucose < 1.11 mmol/L, protein 653 mg/dL, and chloride 113 mmol/L. CSF culture (reported on Day 25) confirmed MDR *K. pneumoniae* (MDR-KP) (Table 1), indicating CNS involvement. On Day 26, ceftazidime/avibactam was stopped, and treatment was adjusted to polymyxin B 500,000 U intravenously every 12 h plus fluconazole 0.2 g intravenously every 12 h (the latter was added for Candida glabrata isolated from urine on Day 23). Follow-up blood cultures were negative on Day 27. A repeat lumbar puncture on Day 35 failed to yield CSF, but only purulent secretion was aspirated from the syringe (Figure 1). The CSF total nucleated cell count had further increased to 44,800 cells/μL and culture (reported on Day 37) again grew MDR *K. pneumoniae*, confirming persistent CNS infection despite ongoing polymyxin B therapy (Tables 1, 2).

**TABLE 2:** Serial CSF and systemic inflammatory parameters during hospitalization.

Parameter (unit)	Normal range	Day 23	Day 35
CSF appearance	Colorless, clear	Pale red, slightly turbid	Purulent
CSF total nucleated cell count (cells/μL)	<10	29,427	44,800
CSF glucose (mmol/L)	2.5–4.5	< 1.11	ND
CSF total protein (mg/dL)	15–45	653	ND
CSF chloride (mmol/L)	120–130	113	ND
CSF opening pressure (mmH_2_O)	80–180	250	ND (dry tap)
Serum WBC (×10^9^/L)	4–10	10.6	10.5
Serum PCT (ng/mL)	<0.10	5.90	0.29
Serum CRP (mg/L)	0–8	66.0	75.0

CSF, cerebrospinal fluid; WBC, white blood cell; PCT, procalcitonin; CRP, C-reactive protein; ND, not determined. Normal ranges are provided for reference. The CSF parameters on Day 35 were not fully obtained because only purulent secretion could be aspirated.

**FIGURE 2:**
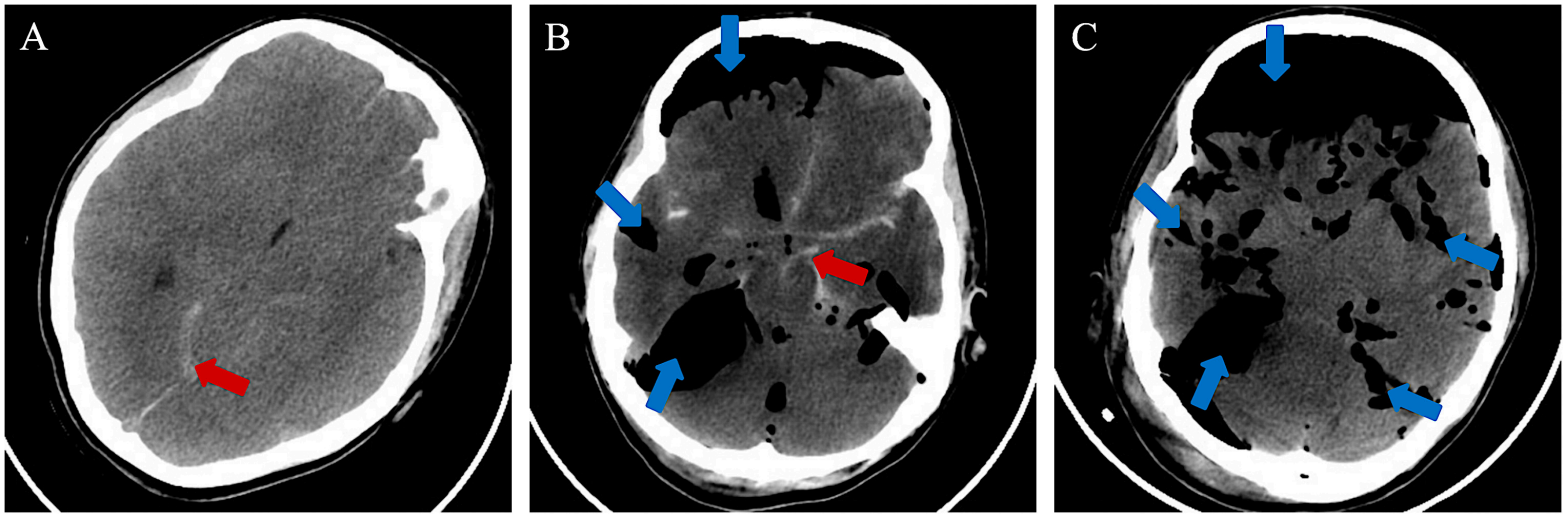
Progression of extensive pneumocephalus on serial cranial CT scans. **(A)** Day 1 (admission): Subarachnoid hemorrhage is noted (red arrow), and no evidence of pneumocephalus or skull fracture is observed. **(B)** Day 19: Significant intracranial gas accumulation is visible, distributed in the frontal, temporal, and occipital lobes (blue arrow); subarachnoid hemorrhage is also noted (red arrow). **(C)** Day 32: Marked progression of pneumocephalus is evident, with extensive gas accumulation throughout the intracranial cavity (blue arrow).

Serial cranial CT scans revealed progressively worsening diffuse cerebral edema, and the patient’s GCS score remained at 3 throughout hospitalization. The initial cranial CT performed on admission showed no evidence of intracranial pneumatosis. By day 19, follow-up CT imaging demonstrated extensive pneumocephalus. Despite prolonged combination antimicrobial therapy, the patient’s pneumocephalus became more extensive (Figure 2), and CSF cultures remained positive for CRKP on day 37, which confirmed sustained involvement of the CNS. The patient’s neurological status showed no improvement, and she ultimately died on day 47.

**FIGURE 3:**
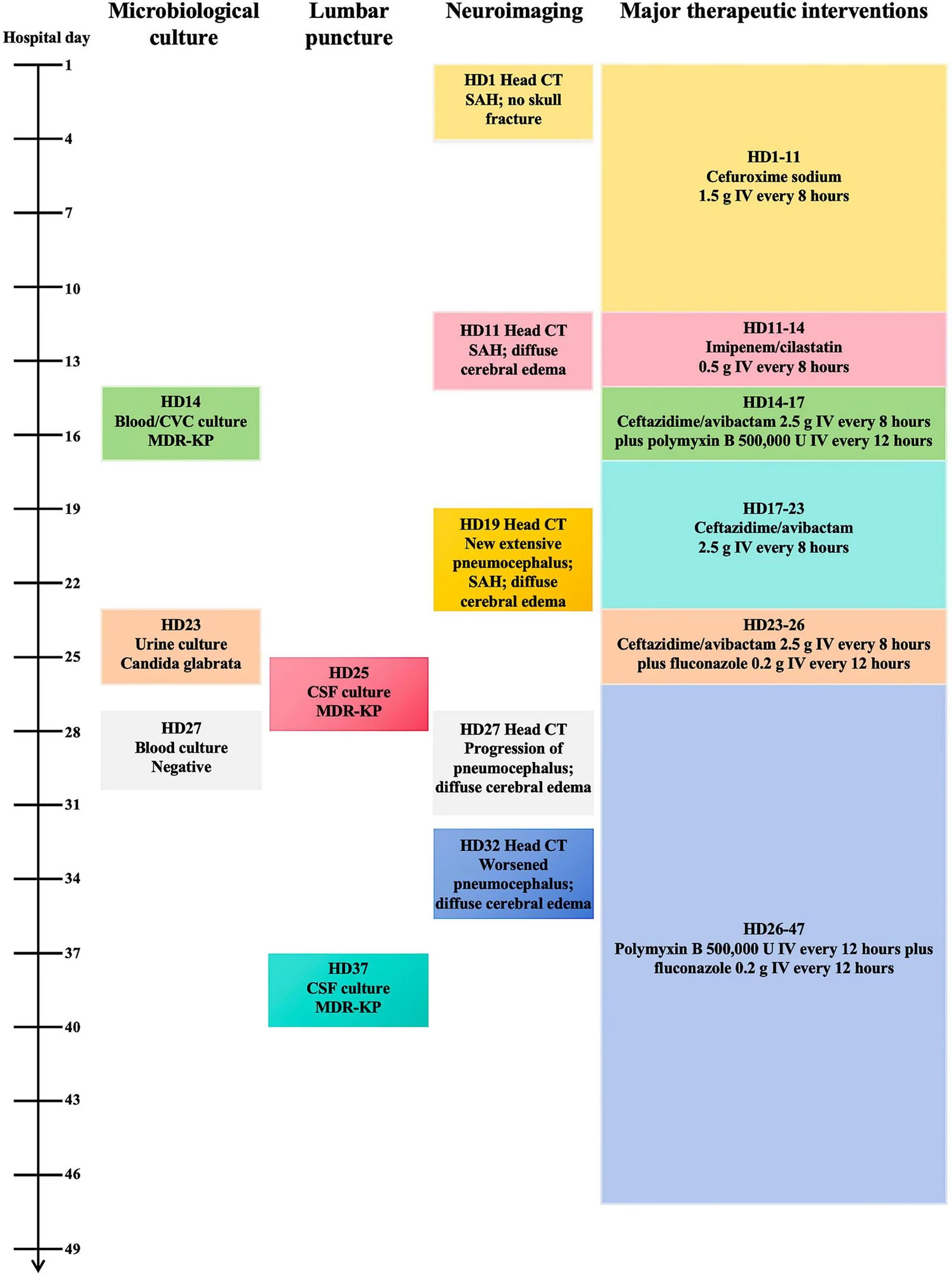
Timeline of microbiological culture, lumbar puncture, neuroimaging, and major therapeutic interventions during hospitalization. HD, hospital day; CT, computed tomography; SAH, subarachnoid hemorrhage; IV, intravenous; MDR-KP, multidrug-resistant *Klebsiella pneumoniae*; CSF, cerebrospinal fluid.

The patient’s complicated hospital course, including the dynamic changes in neuroimaging, microbiological results, and antibiotic adjustments, is summarized in a timeline (Figure 3).

**FIGURE 4:**
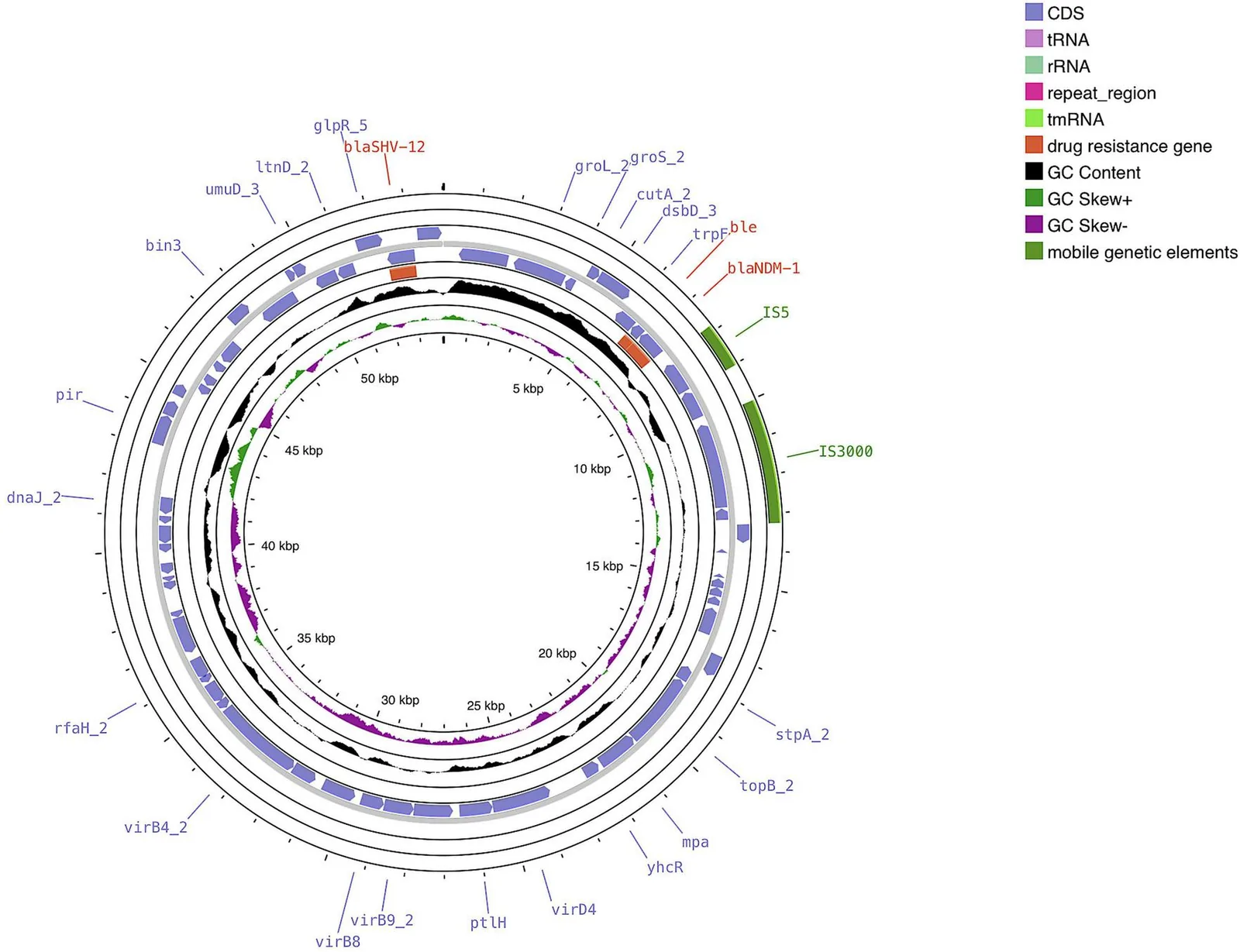
Schematic diagram of the IncX3 plasmid harboring the *bla*NDM-1 gene. The genetic structure of the ∼54 kb plasmid is depicted, showing the location of the *bla*NDM-1 carbapenemase gene (positions 6,750–7,562), flanked downstream by an IS5 family element (positions 7,830–9,024) and an IS3000 (positions 10,032–13,266). Additional annotated features include mobile genetic elements, resistance genes, and other coding sequences.

Retrospective whole-genome sequencing (WGS) and subsequent bioinformatic analyses were performed to precisely characterize the clinical isolates (Figure 4). Multilocus sequence typing (MLST) identified the strain as sequence type ST34, with a KL128 capsular locus and an O3b lipopolysaccharide (LPS) locus. Plasmid replicon typing revealed the coexistence of IncX3 (100% identity, 374/374 bp) and IncFIB(K) (98.93% identity, 560/560 bp) plasmids in both blood and CSF isolates. Analysis of the genetic context revealed that the *bla*NDM-1 gene is located at positions 6,750–7,562 on the 54 kb IncX3 plasmid. Immediately downstream of *bla*NDM-1, an IS5 family element was identified at positions 7,830–9,024 (99.5% identity, 100% coverage), with only approximately 270 bp of intervening sequence. Additionally, an IS3000 was identified at positions 10,032–13,266, approximately 2.5 kb downstream of the *bla*NDM-1, which may facilitate the horizontal transfer of the plasmid. To investigate phylogenetic relatedness, core-genome SNP analysis was performed on the paired isolates, revealing a minimal distance of only 14 SNP differences, strongly suggesting that CNS infection is a direct metastatic complication of previous bacteremia. Resistance gene profiling identified identical acquired resistance gene profiles in both isolates, including the carbapenemase gene *bla*NDM-1 (100% identity), the extended-spectrum β-lactamase (ESBL) gene *bla*SHV-12 (100% identity), the aminoglycoside resistance genes *aph(3”)-lb* and *aph*(6)*-ld*, the tetracycline resistance gene *tet(A)*, the fosfomycin resistance gene *fosA5*, and the efflux pump genes OqxA and OqxB. Virulence gene screening identified only the *ybt* locus (Yersiniabactin, *ybt* 16) in both isolates; hypervirulence-associated genes including *rmpA*, *rmpA2*, *iucA-D* (aerobactin), and *iroB*/*C*/*D*/*N* (salmochelin) were negative.

## 3 Discussion

This case presents a fatal disseminated infection caused by NDM-1-producing CRKP in an 18-year-old female following severe thoracoabdominal trauma. The most clinically challenging features include metastatic CNS infection secondary to bacteremia, confirmed by genomic clonality between blood and CSF isolates; the development of extensive, progressive pneumocephalus without any skull fracture or neurosurgical intervention; and persistent microbiological failure despite optimized combination antimicrobial therapy. Collectively, these features underscore the unique pathophysiology and therapeutic deadlock of NDM-1-producing CRKP CNS infections.

Unlike the trauma-induced hypervirulent *K. pneumoniae* invasive syndrome (KPIS) described by Chen et al. (8), this case involves a classic (non-hypervirulent) but extensively drug-resistant CRKP strain. The primary therapeutic obstacle lies in the pharmacokinetic limitations imposed by the BBB. Although systemic inflammation may partially increase BBB permeability (9), the penetration of most antibiotics remains subtherapeutic for CNS infections (10). NDM-1 hydrolyzes virtually all β-lactams, including carbapenems, leaving only a few agents such as polymyxin B and tigecycline as theoretical options (11). However, the CSF penetration of polymyxin B is poor (approximately 5–11% of serum concentrations) (12), and the concentration of tigecycline in the CNS is also negligible. This profound BBB-restricted drug exposure creates a “pharmacological sanctuary” within the CNS, allowing persistent bacterial replication despite negative follow-up blood cultures. Consequently, management of CNS infection requires consideration of antimicrobial penetration into the CSF. Although intrathecal or intraventricular administration of polymyxins or other agents has been reported for CRKP meningitis (13), these routes were not feasible in this patient due to the rapid neurological deterioration, severe cerebral edema, and critical clinical status.

The occurrence of pneumocephalus without skull fracture or neurosurgery is exceptionally rare, with multiple mechanisms may jointly contributing to this process. First, *K. pneumoniae* is a facultative anaerobe that, under hypoxic conditions, ferments glucose through the mixed-acid pathway, generating CO_2_ and H_2_ (14). In the infected subarachnoid space, local hypoxia triggers this metabolism, leading to intracranial gas accumulation. Although the patient had no history of diabetes mellitus, the acute hyperglycemia (19.00 mmol/L) present upon admission still provided abundant substrate for the fermentation process of *K. pneumoniae*, thereby significantly exacerbating gas production. Second, severe trauma disrupts immune barriers and triggers a pro-inflammatory cytokine storm (IL-6, TNF-α), which paradoxically impairs phagocytosis, and the marked hyperglycaemia further compromises neutrophil function (15). The pathogen enters the bloodstream from a primary focus (wounds), crosses the BBB, and establishes meningitis. The ensuing inflammation causes microvascular thrombosis and focal necrosis (8). Necrotic tissue is poorly oxygenated, creating an anaerobic niche that promotes bacterial proliferation and further fermentative gas production. Third, upon admission, the patient’s cranial CT revealed SAH. SAH itself elevates ICP through a mass effect, which in turn reduces cerebral perfusion and exacerbates local hypoxia (16), thereby promoting anaerobic bacterial proliferation and gas production. Cerebral edema and gas accumulation further aggravate the pressure increase, ultimately forming a vicious cycle. Previously reported cases of pneumocephalus have been predominantly caused by hypervirulent or hypermucoviscous strains and are frequently accompanied by host factors such as diabetes or sepsis, with a subset of patients having skull base defects or a history of surgery (8, 17); however, no pneumocephalus has been reported in CNS infections caused by NDM-producing *K. pneumoniae*. To systematically contextualize our observation, we summarized previously documented cases of NDM-producing CRKP CNS infections, along with their therapeutic strategies and outcomes (Table 3). Concomitant SAH is exceptionally rare; in those cases, SAH was often linked to septic vasculitis (18). Here, SAH is traumatic and precedes infection, yet it synergises with trauma-induced immunosuppression to promote the same vicious cycle. Given that serial imaging examinations revealed no CSF leak, pneumothorax, or pneumomediastinum, occult skull base fracture and ventilator-associated barotrauma are unlikely. Thus, even without an anatomical defect, pneumocephalus can also occur when factors such as traumatic SAH, host immune compromise, bacterial fermentative metabolism, and acute hyperglycemia are present.

**TABLE 3:** Summary of reported cases of NDM-producing *K. pneumoniae* CNS infection.

Patient characteristics	Resistance mechanism	Pneumocephalus	Finalized treatment	Outcome	References
Cerebellar infarction, post-decompressive craniectomy	KPC + NDM co-producing CRKP	No	CZA + aztreonam (simultaneous IV) + topical polymyxin B irrigation	Cured	(30)
SAH, partial skull removal	NDM-1-producing CRKP	No	IV + intrathecal colistin	Cured	(13)
Post-craniectomy	NDM-1-producing CRKP	No	Intrathecal polymyxin E	Cured	(31)
Cerebral hemorrhage, ventricular drainage	NDM-1 + two copies of KPC-2	No	No targeted therapy (discharged AMA before susceptibility results)	Worsened	(32)
Mastoiditis with retrotympanic abscess	OXA-48 + NDM-1 co-producing	No	ATM-AVI full-vial loading dose + continuous infusion	Cured	(22)
Thoracoabdominal trauma, SAH, no skull fracture	NDM-1-producing CRKP	Yes	IV polymyxin B + fluconazole	Dead	Present case

CNS, central nervous system; CRKP, carbapenem-resistant *Klebsiella pneumoniae*; NDM, New Delhi metallo-β-lactamase; KPC, *Klebsiella pneumoniae* carbapenemase; OXA, oxacillinase; CZA, ceftazidime-avibactam; IV, intravenous; ATM-AVI, aztreonam-avibactam; SAH, subarachnoid hemorrhage; AMA, against medical advice.

This case illustrates a multifactorial therapeutic deadlock. Genomic analysis confirmed that the strain carries the *bla*NDM-1 gene, which encodes a metallo-β-lactamase capable of hydrolyzing virtually all β-lactam antibiotics, including carbapenems and ceftazidime-avibactam (19). Consequently, despite multiple antibiotic adjustments during hospitalization based on empirical or culture-guided decisions, the resistant organism could not be covered in the early stage. Of particular note, ceftazidime-avibactam was initially administered according to blood culture results, but this agent is intrinsically inactive against NDM-1-producing isolates; moreover, neither blood nor CSF specimens were subjected to susceptibility testing for this drug. Subsequent intravenous polymyxin B, although a therapeutic option, has extremely poor CNS penetration and fails to achieve bactericidal concentrations in the CSF. In addition, the persistent deterioration of the host’s local microenvironment – with progressive extensive pneumocephalus and tissue hypoxia forming a pro-infection vicious cycle – substantially worsened the prognosis and further hindered antibiotic access to the site of infection. This dual impasse, namely “target ineffectiveness” coupled with “drug scarcity (inadequate central drug concentrations)”, constitutes the primary cause of microbiological failure.

The widespread dissemination of NDM-producing *K. pneumoniae* has rendered clinical anti-infective therapy extremely challenging, yet the emergence and clinical translation of novel agents are gradually reshaping this landscape. The most practically valuable regimen at present is the combination of ceftazidime-avibactam with aztreonam, in which avibactam protects aztreonam from hydrolysis by ESBLs, AmpC, and other β-lactamases, thereby restoring its activity against NDM-1-producing strains; clinical studies have confirmed that this combination achieves microbiological eradication and clinical recovery in severe infections (20). Cefiderocol, as a siderophore cephalosporin, has been highly anticipated, but real-world data indicate that its efficacy may be limited, with NDM-producing strains themselves being associated with poor prognosis; moreover, resistant isolates have emerged even in patients without prior cefiderocol exposure, and the resistance mechanism is closely linked to NDM-1 production, with susceptibility restored by the addition of EDTA or the novel inhibitor xeruborbactam (21) – findings that underscore the need for caution and enhanced surveillance in clinical use. Furthermore, optimization of PK/PD dosing strategies, such as continuous infusion of aztreonam-avibactam or cefiderocol, can significantly improve efficacy without increasing the cost of new drug development and represents a clinically implementable intervention (22, 23). However, in this case, these agents were not administered because of limited availability, delayed identification of the resistance mechanism, and clinical deterioration. Looking ahead, broad-spectrum β-lactamase inhibitors, such as xeruborbactam (a novel inhibitor with activity against class A/B/C/D enzymes, which covers *K. pneumoniae* Carbapenemase, NDM, and oxacillinase), have entered clinical or preclinical development (24), and fragment-based and computer-aided drug design continues to yield novel lead compounds (25). Immunotherapy has achieved breakthrough progress: a vaccine targeting NDM-1 has been shown to trigger humoral immune responses and effectively protect mice from lethal *K. pneumoniae* infection, and human monoclonal antibodies against high-risk capsular types have demonstrated potent prophylactic and cross-protective effects in animal models (26, 27). Phage cocktail therapy, with its capacity for biofilm disruption, offers a promising adjunct to standard antimicrobial treatment (28). Allicin can exert direct antibacterial activity through oxidative stress induction and has the potential to reverse carbapenem resistance via non-competitive inhibition of NDM (29). In summary, the clinical significance of novel agents lies in breaking the previous therapeutic deadlock, yet their application must confront the challenge of rapidly evolving resistance and also face severe challenges in CNS infections, where the BBB’s natural barrier function leads to limited and individually variable CSF penetration. Future efforts should advance the synergistic development of multi-pronged strategies – including novel enzyme inhibitor development, immunotherapy, phage therapy, and individualized dosing optimization – while strengthening global surveillance networks and rapid diagnostic capabilities, to achieve a durable advantage in the battle against this “superbug”.

This case also highlights the indispensable role of infection control and genomic surveillance in preventing nosocomial dissemination of NDM-producing CRKP. Active surveillance of high-risk patients, screening for asymptomatic carriers, strict contact precautions, environmental cleaning, and antimicrobial stewardship are essential to reduce nosocomial transmission. Meanwhile, integrating genome sequencing into routine surveillance enables us to inform timely interventions and to curb the spread of these high-risk pathogens.

Overall, this case offers the following clinical takeaways. First, negative blood cultures do not guarantee negative CSF cultures in CRKP CNS infection. Repeat CSF analysis should be performed to confirm microbiological clearance, even if systemic inflammatory markers have improved or blood cultures have converted to negative. Clinicians should avoid premature de-escalation or discontinuation of anti-CRKP therapy based solely on peripheral blood results. Second, when neuroimaging reveals new or progressive intracranial air in patients with gas-producing microbial infections, clinicians should maintain a high index of suspicion for pneumocephalus, even in the absence of skull fracture or a history of neurosurgery, and promptly initiate a microbiological workup, including CSF culture and molecular assays. Third, when NDM-producing strains are identified or strongly suspected, it is strongly recommended to perform early expanded antimicrobial susceptibility testing and genetic testing, optimize CNS delivery strategies (e.g., intraventricular/intrathecal administration combined with systemic therapy), and explore the early use of novel anti-resistant drugs (e.g., cefiderocol or aztreonam/avibactam).

## 4 Conclusion

This report describes a fatal metastatic CNS infection caused by NDM-1-producing CRKP following severe trauma. WGS confirmed clonal dissemination from bloodstream to CSF, while serial neuroimaging documented progressive extensive pneumocephalus without skull fracture or neurosurgical intervention, representing an exceptionally rare manifestation. Persistent CSF culture positivity despite clearance of bloodstream infection highlights the limitations of current therapeutic strategies, particularly the combined impact of NDM-1-mediated resistance and poor BBB penetration of available antimicrobials. Clinically, repeated CSF microbiological monitoring should not be replaced by negative blood cultures when evaluating treatment response. Early molecular identification of resistance mechanisms, prompt optimization of CNS-active antimicrobial regimens, and consideration of intrathecal or intraventricular therapy when appropriate may improve outcomes. Finally, strengthened genomic surveillance, antimicrobial stewardship, and infection-control measures remain essential to limit the dissemination of NDM-producing CRKP while novel anti-MBL agents and individualized treatment strategies continue to evolve.

## Data Availability

The original contributions presented in the study are included in the article/supplementary material, further inquiries can be directed to the corresponding author.
