# Correction: Extensive pneumocephalus in a fatal central nervous system infection caused by NDM-1–producing carbapenem-resistant *Klebsiella pneumoniae*: a case report

**DOI:** 10.3389/fmed.2026.1992463

**Published:** 2026-09-21

**Authors:** Frontiers Production Office

**Affiliations:** Frontiers Media SA, Lausanne, Switzerland

**Keywords:** *bla*NDM-1 gene, central nervous system infection, genomic characterization, multidrug resistance, pneumocephalus

Author Fangyu Yu was erroneously omitted as corresponding author. The correct corresponding authors are:

¨Qi Li, zjlxlq@163.com

Xiaohong Zhou, zhouxiaohong82@163.com

Fangyu Yu, yufangyu0574@zcmu.edu.cn¨

Affiliation ¨1. Intensive Care Unit, Ningbo Municipal Hospital of Traditional Chinese Medicine (TCM), Affiliated Hospital of Zhejiang Chinese Medical University, Ningbo, China¨ was erroneously given as ¨1. Department of Critical Care Medicine, Ningbo Hospital of Traditional Chinese Medicine, Ningbo, China¨. The affiliation has now been updated.

The original version of this article has been updated.

